# Cytosolic phospholipase A_2 _alpha amplifies early cyclooxygenase-2 expression, oxidative stress and MAP kinase phosphorylation after cerebral ischemia in mice

**DOI:** 10.1186/1742-2094-7-42

**Published:** 2010-07-30

**Authors:** Koji Kishimoto, Rung-Chi Li, Jian Zhang, Judith A Klaus, Kathleen K Kibler, Sylvain Doré, Raymond C Koehler, Adam Sapirstein

**Affiliations:** 1 The Department of Anesthesiology and Critical Care Medicine, Johns Hopkins University School of Medicine, Baltimore, MD, USA

## Abstract

**Background:**

The enzyme cytosolic phospholipase A_2 _alpha (cPLA_2_α) has been implicated in the progression of cerebral injury following ischemia and reperfusion. Previous studies in rodents suggest that cPLA_2_α enhances delayed injury extension and disruption of the blood brain barrier many hours after reperfusion. In this study we investigated the role of cPLA_2_α in early ischemic cerebral injury.

**Methods:**

Middle cerebral artery occlusion (MCAO) was performed on cPLA_2_α^+/+ ^and cPLA_2_α^-/- ^mice for 2 hours followed by 0, 2, or 6 hours of reperfusion. The levels of cPLA_2_α, cyclooxygenase-2, neuronal morphology and reactive oxygen species in the ischemic and contralateral hemispheres were evaluated by light and fluorescent microscopy. PGE_2 _content was compared between genotypes and hemispheres after MCAO and MCAO and 6 hours reperfusion. Regional cerebral blood flow was measured during MCAO and phosphorylation of relevant MAPKs in brain protein homogenates was measured by Western analysis after 6 hours of reperfusion.

**Results:**

Neuronal cPLA_2_α protein increased by 2-fold immediately after MCAO and returned to pre-MCAO levels after 2 hours reperfusion. Neuronal cyclooxygenase-2 induction and PGE_2 _concentration were greater in cPLA_2_α^+/+ ^compared to cPLA_2_α^-/- ^ischemic cortex. Neuronal swelling in ischemic regions was significantly greater in the cPLA_2_α^+/+ ^than in cPLA_2_α^-/- ^brains (+/+: 2.2 ± 0.3 fold vs. -/-: 1.7 ± 0.4 fold increase; *P *< 0.01). The increase in reactive oxygen species following 2 hours of ischemia was also significantly greater in the cPLA_2_α^+/+ ^ischemic core than in cPLA_2_α^-/- ^(+/+: 7.12 ± 1.2 fold vs. -/-: 3.1 ± 1.4 fold; *P *< 0.01). After 6 hours of reperfusion ischemic cortex of cPLA_2_α^+/+^, but not cPLA_2_α^-/-^, had disruption of neuron morphology and decreased PGE_2 _content. Phosphorylation of the MAPKs-p38, ERK 1/2, and MEK 1/2-was significantly greater in cPLA_2_a^+/+ ^than in cPLA_2_α^-/- ^ischemic cortex 6 hours after reperfusion.

**Conclusions:**

These results indicate that cPLA_2_α modulates the earliest molecular and injury responses after cerebral ischemia and have implications for the potential clinical use of cPLA_2_α inhibitors.

## Background

Phospholipase A_2 _(PLA_2_) enzymes hydrolyze free fatty acids from the second position (*sn*-2) of membrane glycerophospholipids and augment neurologic injuries of oxidative stress (reviewed by Muralikrishna [1]). The cytosolic phospholipase A_2_α (cPLA_2_α, also known as PLA_2 _group IVA) is a member of the larger PLA_2 _superfamily and has unique properties that suggest it may regulate formation of eicosanoids in cell-signalling pathways. cPLA_2_α resides in the cytosol but translocates to intracellular membranes in response to physiologic Ca^2+ ^changes [2]. cPLA_2_α has a strong preference for hydrolysis of arachidonic acid (AA); is a major source of regulated, intracellular AA [3]; and is regulated by the protein kinase-dependent phosphorylation of several amino acids [4]. We previously demonstrated that cPLA_2_α is a key effector of neurologic injury following cerebral ischemia and reperfusion (I/R) by showing that cPLA_2_α^-/- ^mice have significantly less stroke injury than do wild-type littermate (+/+) mice after transient regional cerebral ischemia [5]. The presence of cPLA_2_α in neurons [6] and its biochemical properties suggest that it could play a major regulatory role in neurologic signalling in ischemia and other neurologic diseases [7,8].

cPLA_2_α also has a role in the regulation of the downstream enzymes that metabolize AA to the eicosanoids [9,10], which are important mediators of acute and chronic neurologic injury in stroke [11]. The role of COX-2 is particularly well-explored in cerebral I/R and is tightly correlated with cPLA_2_α. Inhibition or gene deletion of COX-2 decreases while COX-2 overexpression enhances neuronal injury following MCAO [12-14]. In mice cPLA_2_α expression appears to be necessary to maintain normal basal and induced expression of COX-2 in the brain [10,15]. cPLA_2_α-derived arachidonic acid is also tightly coupled to the 5-lipoxygenase enzyme [16] and in the gerbil model of global cerebral ischemia 15 minutes of reperfusion caused translocation of 5-LO to the neuron membranes and resulted in increased levels of leukotriene C_4 _[17]. cPLA_2_α amplifies the increase in permeability of the blood-brain barrier after transient ischemia [7], and eicosanoids contribute to the subsequent inflammatory responses [18]. The eicosanoids, particularly prostaglandins (PGs), and AA itself may also contribute directly to the early excitotoxicity that precedes neuroinflammation [19-23]. Our lab and others found that cPLA_2_α can have a direct and early effect on excitotoxicity in vitro [19,24,25].

Here, we examined the effect of transient regional cerebral I/R on cPLA_2_α expression and, in turn, the effect of cPLA_2_α on cyclooxygenase (COX)-2 expression, PGE_2 _levels and reactive oxygen species (ROS) early in the cell-death cascade. We applied transient middle cerebral artery (MCA) occlusion (MCAO) to cPLA_2_α^+/+ ^and cPLA_2_α^-/- ^mice and investigated the effect of cPLA_2_α on early pathways of neurologic injury at 0, 2, and 6 hours of reperfusion. We then correlated cPLA_2_α expression with ROS generation and the phosphorylation of relevant MAPKs. Our results indicate that cPLA_2_α contributes to I/R injury immediately after ischemia.

## Methods

### Materials

Unless otherwise stated, all compounds were purchased from Sigma-Aldrich Company (St. Louis, MO). For immunomicroscopy anti-cPLA_2_α (P505) was purchased from Abcam Inc. (Cambridge, MA). Rabbit anti-cPLA_2_α (N-216) and anti-β-actin antibodies were from Santa Cruz Biotechnology (Santa Cruz, CA). Alexa Fluor 488 and 568 donkey anti-rabbit IgG and NeuroTrace 435/455 Nissl Stain (NT) were purchased from Invitrogen Corporation (Carlsbad, CA).

### Animal Care

All experiments were conducted in accordance with the guidelines of the National Institutes of Health and approved by the Johns Hopkins University Institutional Animal Care and Use Committee. cPLA_2_α^+/- ^mice were a gift from Takao Shimizu (Tokyo University) and were supplied by Jim Clark (Wyeth Pharmaceutical, Cambridge, MA). Mice were housed in a facility with 12-hour diurnal light cycle with free access to food and water. All experimental mice were produced by mating male and female cPLA_2_α^+/- ^mice that were produced and maintained in the C57BL/6J strain.

### Focal Cerebral Ischemia

Transient focal ischemia was induced by MCAO in 10-14-week-old age-matched cPLA_2_α^-/- ^and cPLA_2_α^+/+ ^littermates between 20-28 g. Anesthesia was by spontaneous ventilation of isoflurane in 30% O_2_. A thermostatically controlled warming pad and infrared light were used to maintain the rectal temperature at 37.5 ± 0.5°C during all phases of the surgery. Left-sided MCAO and sham surgery were performed as previously described [5]. After 2 hours of MCAO, the mice were re-anesthetized, the occlusive suture was removed, and the mice were placed in a temperature-controlled environment.

In experiments to measure oxidative stress, 10 mg/kg dihydroethidium (HE) was injected into the jugular vein at the beginning of MCAO. The mice underwent 2-hour MCAO with continuous monitoring of cerebral blood flow (CBF) by laser-Doppler flowmetry, and at 0 or 2 hours of reperfusion, the mice were sacrificed, perfusion fixed, and the brains harvested.

### Regional CBF Assessment

Regional CBF (rCBF) was measured at 60 minutes of ischemia in mice of each genotype and strain, by using [^14^C]-iodoantipyrine ([^14^C]-IAP) autoradiography, as previously described [26]. MCAO was carried out as described above, with additional placement of femoral arterial and venous catheters. At 60 minutes of MCAO, arterial blood pressure, pH, PaCO_2_, and PaO_2 _were measured, and 4 μCi of [^14^C]-IAP was infused intravenously. Coronal brain sections (20 μm) cut on a cryostat were exposed to BioMax film (Kodak, Rochester, NY) for 10 days with [^14^C] standards. From each mouse, we digitized three autoradiographic images from five positions corresponding to coronal sections at +2, +1, 0, -1, and -2 mm from bregma. Regions corresponding to the core anterior cerebral artery (ACA) and MCA territories were outlined in the ipsilateral and contralateral cortex, and signal intensity was determined (ImageJ version 1.36, NIH, Bethesda, MD). rCBF was calculated as previously described [26], and measurements in the three consecutive coronal slices were averaged at each position to yield values of absolute rCBF in each region.

### Fluorescence Microscopy and Quantitative Digital Image Analysis

Following terminal anesthesia, mice were perfused with 3 × weight/volume of normal saline, followed by 4% paraformaldehyde in PBS, and post-fixed in 4% paraformaldehyde and 15% sucrose. For immunofluorescence, 30 μm coronal sections were blocked and quenched with 0.5% H_2_O_2 _in 0.3% normal donkey serum in PBS and incubated with primary antibody overnight at 4°C. The samples were incubated with secondary antibody followed by DAB treatment. Slides were counter-stained with fluorescent Nissl reagent to enable identification of intact neurons by presence of the Nissl substance [27].

Coronal brain sections were examined by confocal microscope LSM510 META (Zeiss, Thornwood, NY). NT, Alexa Fluor 488, and Alexa Fluor 568 were excited with a 405 nm diode laser, a 488 nm Argon laser, and a 561 nm helium-neon laser, respectively. Emission was detected through 420-480-nm, 505-530-nm, and 565-595-nm band-pass filters, respectively. HE was visualized by excitation at 561 nm and emission at 610 nm. An investigator blinded to genotype and hemisphere used Image J software to measure total cPLA_2_α fluorescence in low magnification (10×) images obtained from representative brain sections of cPLA_2_α^+/+ ^and cPLA_2_α^-/- ^mice.

For high resolution analysis, two representative images in the cortical subfield of interest were acquired from each of three brain sections per mouse, and two *z*-planes of ~2 μm optical thickness separated by 8 μm were sampled. Fluorescence threshold levels were set to allow for recognition of individual neurons in slices without signal saturation and were constant for analysis of all slices. The anatomical regions corresponding to the ischemic core and penumbra were identified in fluorescent Nissl-stained sections. Fluorescence above the threshold was measured in 120-130 neurons for each mouse in non-overlapping, randomly chosen regions in photomicrographs obtained using 100× magnification. Total pixel area was normalized to the total area analyzed and number of neurons and expressed in arbitrary units.

### Immunoblotting

For Western analysis, primary antibodies included COX-2 (1:1000, Cayman Chemical Co. Ann Arbor, MI), cPLA_2_α (1:500), phospho-cPLA_2_α (1:500), ERK1/2 and phospho-ERK1/2 (1:1000), MEK1/2 and phospho-MEK1/2 (1:1000), p38 MAPK and phospho-p38 MAPK (1:1000) (all from Cell Signalling Technology, Inc. Danvers, MA). Protein samples were separated by electrophoresis and transferred to PVDF membranes. Immunocomplexes were visualized by enhanced chemiluminescence detection (Amersham Life Science).

Subcellular fractions were prepared from brain tissue homogenized by Dounce (20 strokes) in 10× v/w of ice-cold lysis buffer (2 mM EGTA in PBS with protease inhibitor), and 1/10 volume of benzonase solution (1:50 dilution). The samples were gently shaken on ice for 20 minutes and centrifuged at 800 × *g *for 10 minutes at 4°C. Supernatant volumes of 100 μl were centrifuged at 100,000 × *g *for 45 min at 4°C. The supernatants contained the cytosolic fraction. The pelleted nuclear fraction was resuspended in 0.7 w/v CHAPS lysis buffer, sonicated for 10 seconds and incubated on ice for 30 minutes. Protein concentrations were measured by the modified Bradford assay. Cell lysate proteins (25 μg per sample) were electrophoretically resolved on 4-15% polyacrylamide Tris-HCl gradient gels (BioRad, Hercules, CA) and transferred to PVDF membranes. Each membrane was probed and stripped sequentially for phospho-cPLA_2_α, cPLA_2_α, and β-actin. For routine immunodetection of proteins cortical hemispheres were homogenized in 5 × v/w buffer, and 10 μg of crude homogenate was used for SDS-PAGE.

### Prostaglandin E_2 _(PGE_2_) Enzyme Immunoassay

Cortical tissue was weighed and homogenized by polytron in 10 μl/mg wet tissue of ice-cold PBS with 10 μg/ml indomethacin and incubated on ice for 10 min. The homogenate solution was brought to 40% volume aqueous ethanol and acidified with glacial acetic acid to pH 3.0, incubated for 5 min at room temperature, and centrifuged at 2,500 × *g *for 10 min. The supernatant was applied to a conditioned Oasis HLB column (Waters Corp., Milford, MA), washed with 0.03% formic acid, followed by 15% aqueous ethanol/0.03% formic acid followed by petroleum ether. PGs were eluted with ethyl acetate and evaporated to dryness under nitrogen. The eluant was dissolved in 300 μL assay buffer, and PGE_2 _concentration was determined by ELISA according to the manufacturer's instructions (Assay Designs, Ann Arbor, MI.). For each extraction and ELISA the results were normalized within the group to account for variation in the efficiency of lipid extraction.

### Statistical Analysis

Assays that required multiple samples from a single mouse were analyzed by averaging the intra-mouse samples and then performing statistical annalysis between individuals. For studies in which multiple time points were compared across genotypes and hemispheres analysis was performed by repeated measures ANOVA and post-hoc comparison between genotypes was made with the Newman-Keuls test. Comparison of relative PGE_2 _concentrations following MCAO between genotypes and hemispheres was conducted with 2-way ANOVA followed by Bonferroni testing between the genotypes using GraphPad Prism version 5.03 (GraphPad Software, San Diego California). Densitometry analysis was by paired t-tests. For all procedures; *P *< 0.05 was considered statistically significant. Data are expressed as mean ± s.d.

## Results

**Figure 1:**
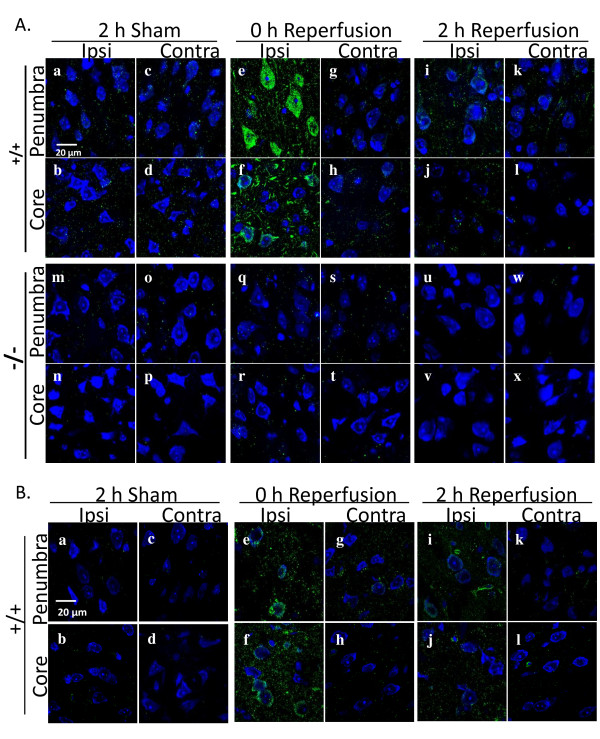
**cPLA_2_α is increased by ischemia in cPLA_2_α^+/+ ^neurons**. **A: **Immunofluorescence of cPLA_2_α (green) and fluorescent Nissl dye (blue) in brain slices from cPLA_2_α^+/+ ^and cPLA_2_α^-/- ^mice after sham surgery, 2-hour MCAO, or 2-hour MCAO and 2-hour reperfusion. Images from the ischemic core and penumbra regions from the ipsilateral and contralateral hemispheres of each genotype are shown. **B: **Immunofluorescence of Ser505-phosphorylated cPLA_2_α (green) in cPLA_2_α^+/+ ^mice subjected to sham surgery (a-d), 2 hours of MCAO (e-h) or 2 hours of MCAO and 2 hours of reperfusion (i-l). Fluorescent Nissl staining of neurons is in blue. Representative images, n = 3 mice of each genotype. Ipsi, ipsilateral; Contra, contralateral.

To examine the effect of cPLA_2_α expression on the cascade of molecular and cellular events *in vivo *following cerebral I/R, we subjected cPLA_2_α^+/+ ^and cPLA_2_α^-/- ^mice to 2 hours of MCAO followed by no (0), 2, or 6 hours of reperfusion and examined the expression of cPLA_2_α using immunofluorescence coupled with Nissl staining. We observed a substantial increase in the level of cPLA_2_α staining in the cPLA_2_α^+/+ ^mice after 2 hours of MCAO and no reperfusion. The averaged cPLA_2_α fluorescence intensity in cPLA_2_α^+/+ ^ischemic hemispheres was 1.9 fold greater than that in contralateral hemispheres (*P *< 0.01). As expected, the nonspecific staining in cPLA_2_α^-/- ^hemispheres was barely detectable and was not altered by ischemia. We then used high resolution imaging to characterize the cellular expression patterns of cPLA_2_α that follow MCAO in the ischemic core and penumbra regions. We observed a very low level of cPLA_2_α immunofluorescence in cPLA_2_α^+/+ ^mice after sham surgery (Figure 1A a-d). After 2 hours of ischemia, the immunofluorescence was markedly increased in the neurons and non-neuronal cells of the ischemic hemisphere (Figure 1A e-f) but was unchanged in the contralateral hemisphere (Figure 1A g-h). However, after 2 hours of reperfusion, cPLA_2_α was substantially lower in the neurons of the penumbra (Figure 1A i) and almost absent in the neurons of the ischemic zone (Figure 1A j). Nissl staining suggests loss of neurons in the ischemic core after 2 hours of reperfusion (Figure 1A j). Six hours after reperfusion, cPLA_2_α immunofluorescence could not be distinguished from that of sham-operated mice (data not shown). The cPLA_2_α^-/- ^mice had minimal, nonspecific background staining (Figure 1A m-x). Phosphorylated cPLA_2_α also showed a marked increase in cPLA2α^+/+ ^brain after 2 hours of ischemia and then decreased along a time course similar to that of unphosphorylated cPLA_2_α (Figure 1B).

**Figure 2:**
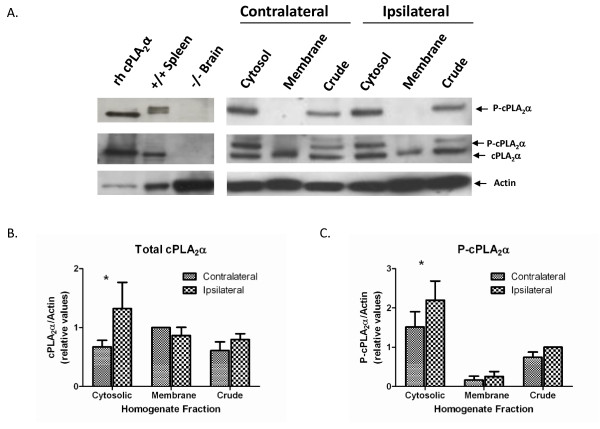
**cPLA_2_α is increased in the ischemic hemisphere following focal ischemia**. **A: **A representative Western blot of cPLA_2_α^+/+ ^brain protein fractions (25 μg each lane) from the non-ischemic (contralateral) and ischemic (ipsilateral) hemispheres. The same membrane was exposed to antibody and developed sequentially for phospho-cPLA_2_α, cPLA_2_α, and actin. The panel on the left represents positive controls-recombinant human cPLA_2_α (rh cPLA_2_α) and mouse spleen protein-and a negative control, cPLA_2_α^-/- ^brain protein. The antibody directed against cPLA_2_α recognizes both the phosphorylated and nonphosphorylated cPLA_2_α. **B, C: **Densitometry analysis of Western blots for (B) total cPLA_2_α (n = 5 experiments) and (C) phospho-cPLA_2_α (n = 5 experiments). * *P *< 0.05 ipsilateral compared to contralateral.

To validate the results of the immunofluorescence experiments, cPLA_2_α^+/+ ^mice were subjected to 2-hour MCAO and no reperfusion, or sham operation. Following euthanasia the ipsilateral and contralateral cortices were harvested for protein extraction. We performed a subcellular fractionation on the cortical proteins and subjected these to Western blot analysis using anti-cPLA_2_α and anti-phospho-cPLA_2_α antibodies. The anti-cPLA_2_α antibody recognizes both the phosphorylated and unphosphorylated forms of cPLA_2_α and this leads to the formation of a doublet on immunoblot. The upper band of this doublet is the phospho-cPLA_2_α form and this is confirmed with the anti-phospho-cPLA_2_α antibody. Consistent with the immunofluorescence findings, 2 hours of ischemia increased total and phospho-cPLA_2_α in the ipsilateral cytosolic fraction as compared to the contralateral (non-ischemic) cytosolic fraction (Figure 2). Expression levels of total and phospho-cPLA_2_α in the membrane fraction did not differ between the ipsilateral and contralateral hemispheres. This indicates that cPLA_2_α is not associated with cellular membranes following 2 hours of MCAO.

**Figure 3:**
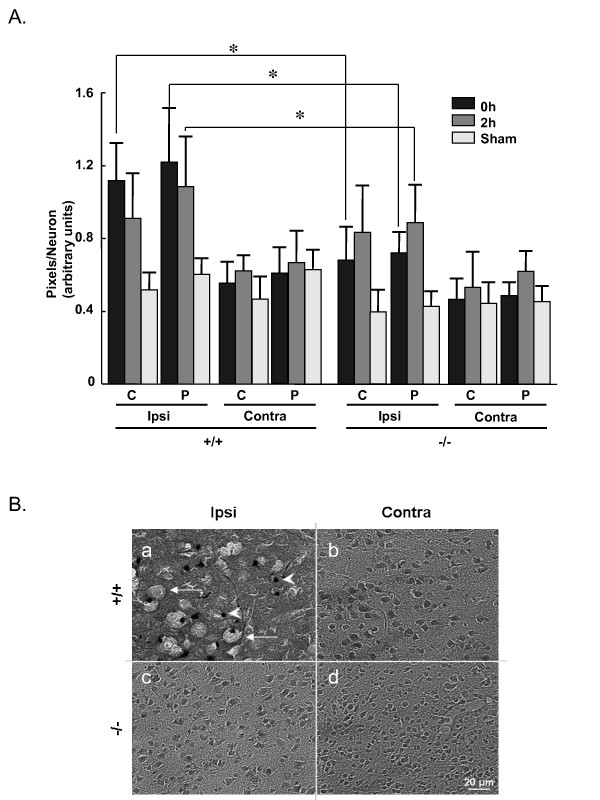
**Neurons in the ischemic core of the cPLA_2_α^-/- ^mouse are protected from injury**. **A: **Neuronal swelling was measured by threshold densitometry in brains from cPLA_2_α^+/+ ^and cPLA_2_α^-/- ^mice after 2-hour MCAO, 2-hour MCAO and 2 hours of reperfusion, or sham surgery; n = 3 mice/condition; * *P *< 0.01, unpaired *t*-test comparing cPLA_2_α^+/+ ^to -/- at same condition. **B: **Nissl staining of cPLA_2_α^+/+ ^and cPLA_2_α^-/- ^brains after 2-hour MCAO and 6 hours of reperfusion. Vacuolated neurons (arrows) and nuclear condensation (arrowheads) are seen in the ischemic core of cPLA_2_α^+/+ ^mice. Ipsi, ipsilateral; Contra, contralateral; C, ischemic core; P, penumbra.

Nissl staining illustrated that I/R caused much greater disruption of cortical pyramidal neuron morphology in cPLA_2_α^+/+ ^mice than in cPLA_2_α^-/- ^mice. Neurons in the core and penumbra regions were enlarged immediately after 2-hour ischemia (0 hours of reperfusion) and after 2 hours of reperfusion (Figure 3A and Table 1). The expression of cPLA_2_α was associated with greater neuronal swelling at both time points. After 6 hours of reperfusion, neuronal structure in the cPLA_2_α^+/+ ^ipsilateral hemisphere was almost completely disrupted with a dramatic reduction in the number of neurons (Figure 3B, a). The structure and number of neurons in cPLA_2_α^-/- ^mouse brains, however, remained intact (Figure 3B, c).

**Table 1:** cPLA2α^+/+ ^and -/- neuron size after MCAO, reperfusion or sham surgery

	cPLA_2_α^+/+ ^Neuron Area (arbitrary units)				cPLA_2_α^-/- ^Neuron Area (arbitrary units)			
	Ipsilateral		Contralateral		Ipsilateral		Contralateral	
	Core	Penumbra	Core	Penumbra	Core	Penumbra	Core	Penumbra
**0 h Reperfuse**	1.12 ± 0.21*	1.22 ± 0.30 *	0.55 ± 0.12	0.61 ± 0.15	0.68 ± 0.19	0.72 ± 0.12	0.46 ± 0.12	0.49 ± 0.08
**2 h Reperfuse**	0.91 ± 0.25	1.08 ± 0.28 *	0.62 ± 0.09	0.67 ± 0.18	0.83 ± 0.26	0.89 ± 0.21	0.53 ± 0.2	0.62 ± 0.11
**Sham**	0.52 ± 0.09	0.60 ± 0.09	0.47 ± 0.13	0.63 ± 0.11	0.40 ± 0.13	0.43 ± 0.09	0.44 ± 0.12	0.45 ± 0.09

Relative neuron size was determined in fluorescent Nissl-stained brain sections by threshold densitometry. Sections from the ipsilateral and contralateral hemispheres were matched for position in mice that were subjected to 2 hours MCAO, MCAO and 2 hours reperfusion or sham surgery. The data are an index of the average number of pixels in a neuron for each condition. * *P *< 0.01 for +/+ as compared to -/- of the same condition.

**Figure 4:**
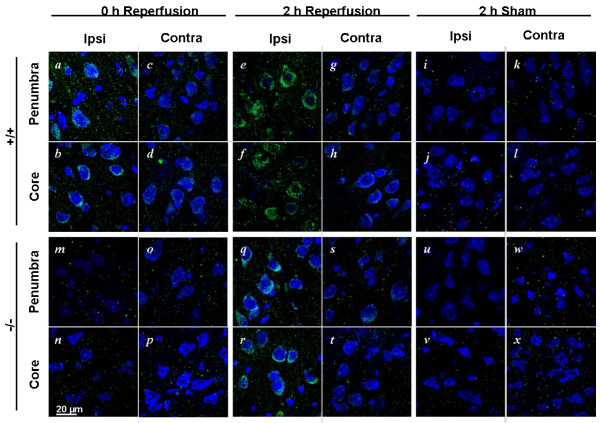
**cPLA_2_α regulates post-ischemic COX-2 levels**. Immunofluorescence of COX-2 (green) and fluorescent Nissl dye (blue) in brain slices from cPLA_2_α^+/+ ^and cPLA_2_α^-/- ^mice after 2-hour MCAO, 2-hour MCAO and 2-hour reperfusion, or sham surgery. Images of the ischemic core and penumbra regions from the ipsilateral and contralateral hemispheres of each genotype are shown. Representative images, n = 3 mice of each genotype. Ipsi, ipsilateral; Contra, contralateral.

cPLA_2_α regulates COX-2 expression in the brain [10,15] and nonspecific PLA_2 _blockade prevents COX-2 induction after transient focal ischemia [28]. We examined the effect of cPLA_2_α deletion on COX-2 expression after I/R. In the ipsilateral cortices of cPLA_2_α^+/+ ^mice, COX-2 immunofluorescence was substantially greater than that in sham-operated controls immediately after ischemia (Figure 4A a-b compared to i-j) and increased further 2 hours after reperfusion (Figure 4A e-f). In contrast, COX-2 was not elevated in the ipsilateral cortex of cPLA_2_α^-/- ^mice (Figure 4A m-n) and was only slightly increased after 2 hours of reperfusion (Figure 4A q-r).

**Figure 5:**
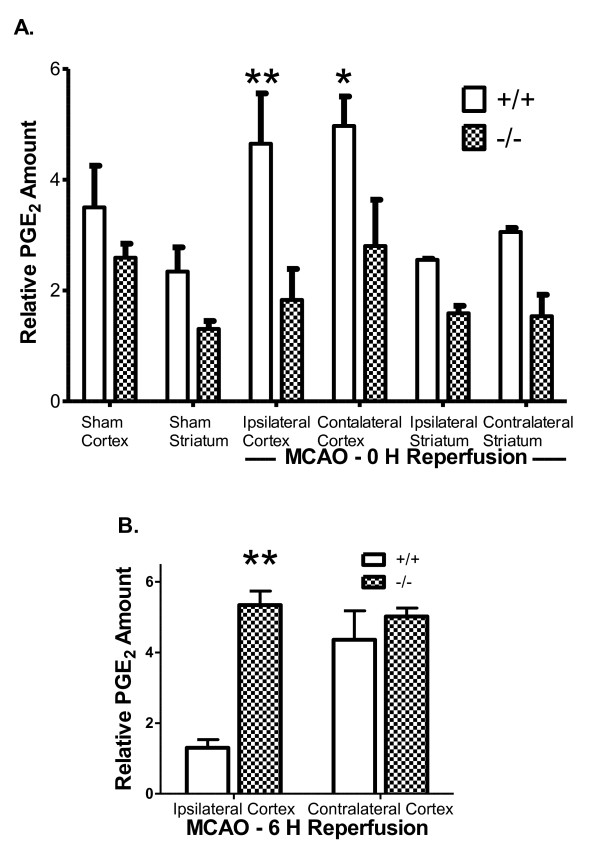
**Concentration of cortical PGE_2 _following MCAO and reperfusion is dependent on cPLA_2_α**. PGE_2 _was measured in cortical homogenate from the ipsilateral and contralateral hemispheres after **A: **2 hours MCAO or sham operation and **B: **2 hours of MCAO and 6 hours reperfusion. Results are expressed as normalized for each lipid extraction and assay. * *P *< 0.05; ** *P *< 0.01 n = 3 - 4 each condition.

PGE_2 _is produced by the coordinated enzymatic activities of COX and the PGE synthases upon AA. Previous studies have demonstrated that PGE_2 _levels are elevated following MCAO in the rat hippocampus [29]. We compared the levels of PGE_2 _in the cortex of cPLA_2_α^+/+ ^and -/- mice immediately following 2 hours of ischemia and no reperfusion (Figure 5A) or after 6 hours of reperfusion (Figure 5B). In agreement with previous results there was no significant difference between basal PGE_2 _levels in the cPLA_2_α^+/+ ^and -/- cortex [10]. However 2 hours of MCAO caused a significant increase in the PGE_2 _concentration of both the contralateral and ipsilateral cPLA_2_α^+/+ ^cortices. In contrast the levels of PGE_2 _were not changed by ischemia in the cPLA_2_α^-/- ^cortex. After 6 hours of reperfusion the concentration of PGE_2 _in ischemic cPLA_2_α^+/+ ^cortex was significantly lower than in cPLA_2_α^-/- ^cortex or in the contralateral cortex of either genotype (Figure 5B).

**Figure 6:**
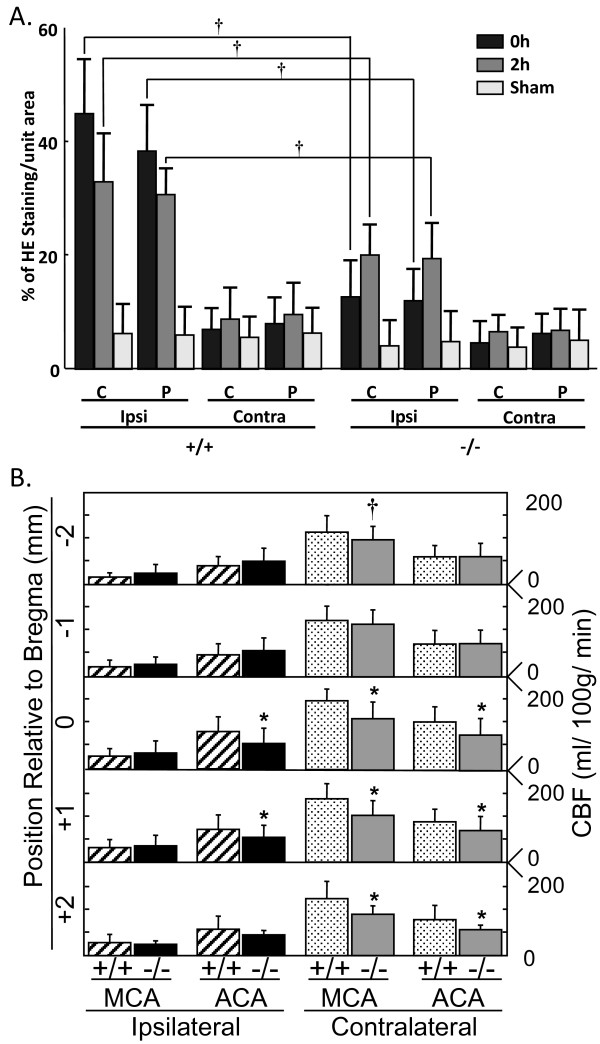
**Oxidative stress is greater in cPLA_2_α^+/+ ^than in cPLA_2_α^-/- ^mice after MCAO**. **A: **ROS were measured in cPLA_2_α^+/+ ^and cPLA_2_α^-/- ^mice that were injected with dihydroethidium (HE) immediately before MCAO or after sham surgery. The intensity of HE staining in brain sections from the indicated time points was measured by densitometry. Ipsi, ipsilateral; Contra, contralateral; n = 3 mice/condition with 120-130 neurons counted/mouse; † *P *< 0.01. **B: **CBF was measured in mice by [^14^C]-IAP autoradiography in the ACA and MCA territories of each cortex at 60 minutes of MCAO. The left axis shows the position (in mm) of each region relative to bregma, and the right axis shows the absolute rCBF (ml/100 g/min); 24 slices from n = 4 mice/group. * *P *< 0.01 and † *P *< 0.05 as compared to the same region in cPLA_2_α^+/+ ^mice.

We also evaluated the role of cPLA_2_α expression in the generation of ROS using the fluorescent probe HE. The increase in ROS in the ischemic hemisphere of cPLA_2_α^+/+ ^mice was significantly greater than in the cPLA_2_α^-/- ^mice following ischemia without reperfusion (Figure 6A, 0 h) (+/+: 7.12 ± 1.2 fold increase vs. -/-: 3.10 ± 1.4 fold increase, *P *< 0.01) and also 2 hours after ischemia (Figure 6A and Table 2). Levels of ROS in the contralateral hemispheres were not different from levels in sham-operated mice.

**Table 2:** Oxidative stress in cPLA_2_α^+/+ ^and -/- cortex after MCAO, MCAO and reperfusion, or sham surgery

	cPLA_2_α^+/+ ^HE area (relative units)				cPLA_2_α^-/- ^HE area (relative units)			
	Ipsilateral		Contralateral		Ipsilateral		Contralateral	
	Core	Penumbra	Core	Penumbra	Core	Penumbra	Core	Penumbra
**0 h Reperfuse**	45.02 ± 9.58†	38.36 ± 8.53†	6.99 ± 3.88	8.07 ± 4.70	12.76 ± 6.83	12.06 ± 5.85	4.66 ± 4.17	6.27 ± 3.83
**2 h Reperfuse**	33.01 ± 8.59†	30.70 ± 5.14†	8.82 ± 5.96	9.62 ± 5.95	20..09 ± 5.50	19.47 ± 6.72	6.60 ± 2.98	6.80 ± 4.09
**Sham**	6.32 ± 5.46	6.03 ± 5.03	5.63 ± 3.71	6.40 ± 4.65	4.11 ± 4.48	4.88 ± 5.43	3.85 ± 3.54	5.05 ± 5.67

Oxidative stress was measured in the cortex of cPLA_2_α^+/+ ^and -/- brains using micro-fluorescence and threshold densitometry. At the beginning of MCAO or sham surgery 10 mg/kg dihydroethedium (HE) was injected into the relative jugular vein of each mouse. The intensity of HE staining in each region is normalized for area and expressed in relative units. n = 3 mice per condition with analysis of high resolution confocal z-plane images from each mouse as described in the text. †, *P *< 0.01 cPLA_2_α^+/+ ^compared to -/- at the same condition.

To determine if differences in ROS levels between cPLA_2_α^+/+ ^and cPLA_2_α^-/- ^mice resulted from differences in the vascular responses during ischemia, rCBF was measured by the technique of [^14^C]-IAP injection. The cortical regions corresponding to the ACA and MCA were demarcated in coronal brain sections. MCAO caused a significant reduction of blood flow in both the ACA and MCA territories, relative to the contralateral sides in each genotype (Figure 6B). CBF was slightly lower in the ipsilateral ACA territory in the anterior region of the cPLA_2_α^-/- ^brain than in the corresponding region of the cPLA_2_α^+/+ ^brain. A similar level of ACA blood flow reduction was measured in the anterior regions of the contralateral cortex of cPLA_2_α^-/- ^mice. Therefore, differences in rCBF between the genotypes during ischemia did not account for the decrease in HE intensity, COX-2, or neuronal loss in the cPLA_2_α^-/- ^mice.

**Figure 7:**
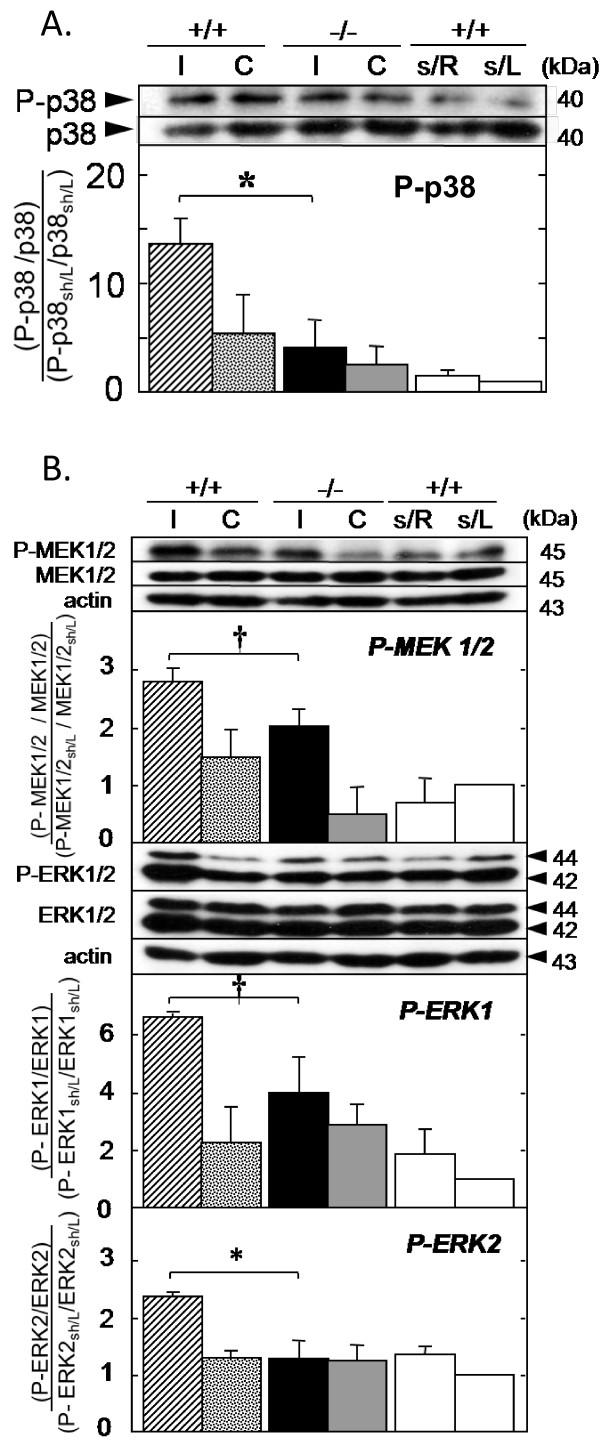
**Activation of MAPK following reperfusion is cPLA2α-dependent**. **A: **The relative expression of phosphorylated p38 MAPK (P-p38) was measured in cPLA_2_α^+/+ ^and cPLA_2_α^-/- ^after MCAO and 6 hours of reperfusion. Representative Western blots of a single membrane are shown above the quantification from three experiments. P-p38 levels were quantified by densitometry and are expressed as the ratio of P-p38 to total p38 relative to the ratio of P-p38 to total p38 in sham-operated cPLA_2_α^+/+ ^mice. **B: **Representative Western blots of MEK1/2 and ERK1/2 are shown. Relative levels of phosphorylated MEK1/2 (P-MEK1/2) and ERK1/2 (P-ERK1/2) were calculated as described in **A**. In all cases, actin was used as a control for loading. I, ipsilateral; C, contralateral; s/R and s/L, right and left side cortices of sham-operated mice (n = 3 from 3 mice). # *P *< 0.01; † *P *< 0.05.

Activation of protein kinases, including p38 MAPK, MEK1/2, and ERK1/2, has been implicated in neuronal death and survival following cerebral reperfusion [30] and has been associated with cPLA_2_α activity [7]. MCAO followed by 6 hour reperfusion caused increased levels of phosphorylated p38 MAPK that were significantly higher in the ischemic hemisphere of the cPLA_2_α^+/+ ^mice than in cPLA_2_α^-/- ^mice (Figure 7A). Phosphorylation of MEK1/2 and ERK1/2 proteins was also significantly greater in the ischemic hemispheres of cPLA_2_α^+/+ ^than cPLA_2_α^-/- ^mice (Figure 7B).

## Discussion

The cPLA_2_α amplifies neural injury in animal models of acute and chronic injury, and it is likely that it modulates direct injury and inflammatory pathways [5,31,32]. In our previous study, we postulated that reduction of infarct size in cPLA_2_α^-/- ^mice resulted from a reduction in the delayed extension of injury into the penumbra [5]. In the current study, we measured cPLA_2_α expression after I/R and compared COX-2 expression, PGE_2 _levels and ROS formation in the brains of cPLA_2_α^+/+ ^and cPLA_2_α^-/- ^mice at different times after reperfusion (0-6 hours). Importantly, these early time points precede the largest influx of circulating inflammatory cells and blood-brain barrier disruption in experimental stroke [7,33]. Our results show for the first time that ischemia induces cPLA_2_α expression and this is correlated with COX-2 expression and formation of ROS (as indicated by HE intensity). Taken together, our results indicate that cPLA_2_α plays an important role *in vivo *in the early toxic events after I/R.

The changes in the levels of cPLA_2_α protein that we observed following MCAO, while significant, were small. The reasons for this include the fact that the abundance of cPLA_2_α compared to other PLA_2_s in the brain is small [34]. Secondly the proteins used for Western analysis are prepared from tissue samples that include regions where cPLA_2_α levels may not have changed. This will reduce the observed effect of ischemia on cPLA_2_α expression. Previously published data support the neuronal induction of cPLA_2_α following ischemia. Alexandrov and colleagues [35] identified a hypoxia-sensitive domain in the 5'-untranslated region of the human cPLA_2_α gene that induces cPLA_2_α mRNA in brain microvascular endothelial cells. Numerous studies have reported cPLA_2_α expression in glial cells [36] and mRNA expression in neurons [6], and a recent study showed that cPLA_2_α is expressed in neurons in a mouse model of Alzheimer's disease [8]. After transient global ischemia, late induction of cPLA_2_α was found only in glial cells [37]. Other investigators have noted an early increase in PLA_2 _activity minutes after global cerebral I/R [38]. A rat model of transient cerebral ischemia showed that cPLA_2_α activity increased 1 day after reperfusion but that the levels of protein and phospho-cPLA_2_α did not increase until 3 days after reperfusion [7]. Changes in cPLA_2_α that occur hours to days following ischemia may be related to secondary injury and inflammation.

In cell culture models, chemical anoxia [39] and increased intracellular calcium [40] cause cPLA_2_α to translocate to nuclear and other membranes. In our immunofluorescence and subcellular fractionation experiments ischemia did not cause translocation of cPLA_2_α to membranes. There are several potential explanations for the lack of cPLA_2_α membrane association. In the gerbil global ischemia model, 5-LO did not translocate to the nucleus until minutes after reperfusion [17]. Similarly, reoxygenation following ischemia appears to be a major determinant of intracellular Ca^2+ ^flux (reviewed by Szydlowska and Tymianski [41]). Thus, it is possible that cPLA_2_α translocates to cellular membranes minutes after reperfusion. Further experiments examining the immediate reperfusion period will be necessary to delineate the intracellular signalling events of cPLA_2_α activation and translocation in neurons.

How could cPLA_2_α impact neuronal injury at times that precede classical neuroinflammation? Mechanisms including increased PG synthesis and action, modulation of excitotoxic responses and increased ROS stress have been postulated.

The cPLA_2_α-associated increase in PGE_2 _levels in cPLA_2_α^+/+ ^cortex following MCAO are consistent with these postulates. In the ischemic core, we found that neuronal COX-2 induction was delayed and decreased in the cPLA_2_α^-/- ^mice and that cPLA_2_α^-/- ^neuronal architecture was preserved. Basal cerebral COX-2 activity and protein levels are significantly decreased in cPLA_2_α^-/- ^mice [15], and we previously found that cortical COX-2 and PGE_2 _responses to lipopolysaccharide were attenuated in cPLA_2_α^-/- ^mice [10]. Systemic effects of MCAO may explain the increase in PGE_2 _in both hemispheres following unilateral MCAO. Work from several laboratories indicates that PGE_2 _signalling through the EP1 or EP3 receptors exacerbates early stroke injury [22,42-44], perhaps through increased calcium responses [23]. Kunz and colleagues observed that early morphologic changes in neurons represented terminal injury and showed that such injury correlated with COX-2 expression and was dependent on PGE_2 _and EP1 receptors but not on formation of ROS [20]. Indeed, Miettinen and co-authors used a nonspecific PLA_2 _inhibitor to ameliorate both injury and COX-2 induction following transient MCAO and suggested that neurons that express cPLA_2_α are more sensitive to ischemic damage [28]. The coordinated neuronal activities of cPLA_2_α and COX-2 generate eicosanoids after ischemia which are likely coupled to neuronal G-protein-coupled receptors in a toxic cascade.

Metabolism of AA results in the generation of superoxide, and a detailed kinetic analysis of brain lipids showed decreased AA incorporation in phospholipids of cPLA_2_α^-/- ^mouse brains [45]. Targeting cPLA_2_α to the endoplasmic reticulum exacerbates oxidative stress in cultured cells [46]. In the rat, transient global ischemia causes a rapid release of free fatty acids from the cortex that correlates with an increase in cPLA_2_α activity during the period of ischemia [47]. It is likely that the ischemic cortex of a cPLA_2_α^-/- ^mouse has less stimulated AA release and therefore less ROS formation. cPLA_2_α may contribute to ROS formation through an AA-dependent, COX-2 independent pathway.

AA released by cPLA_2_α also has the potential to significantly affect glutamate excitotoxicity. The application of a cPLA_2_α inhibitor to cultured hippocampus significantly protected pyramidal neurons from oxygen-glucose deprivation [24], and PLA_2 _inhibitors reduced the release of excitatory amino acids from the cortical surface following 4-vessel occlusion in the rat [48]. In cultured neurons, AA amplifies the calcium response to NMDA stimulation [21]. Additionally, we reported that cPLA_2_α activity causes increased neuronal death, rapid broadening of action potentials, and increased Ca^2+ ^transients following NMDA exposure in the CA1 neurons of acute hippocampal slices [19]. Therefore, it is possible that I/R activates cPLA_2_α, causing excessive release of AA, which amplifies the processes of excitotoxicity.

The interaction between cPLA_2_α and the MAP kinase pathways have potential importance in brain I/R injury. Our data demonstrate that cPLA_2_α enhances ROS formation by MCAO (Figure 7) while others have shown that oxidative stress in mouse embryonic stem cells causes MAPK dependent phosphorylation of cPLA_2_α [49]. This interaction has the potential to form a positive feedback loop in which cPLA_2_α-dependent ROS increase kinase activation which leads to further cPLA_2_α activation. We examined the state of MAPK phosphorylation after 6 hours of reperfusion for several reasons. First, our results demonstrated neuronal injury at this time. Second, Alessandrini and colleagues [30] showed that *in vivo *cerebral I/R activates these kinases and that inhibition of MEKs is neuroprotective. Third, similar to our results, 2 hours of MCAO followed by reperfusion in the rat causes phosphorylation of ERK1/2 in both the ipsilateral and contralateral cortex after 6 hours of reperfusion [50]. Lastly, Nito et al. demonstrated that p38 phosphorylation and activity peaked following 2 hours MCAO and 6 hours reperfusion [7]. A reduction in cPLA_2_α-dependent ROS may explain why p38 MAPK and MEK1/2-ERK1/2 proteins are less phosphorylated in the cPLA_2_α^-/- ^brain (Figure 7). Oxidative stress activates p38 MAPK in neurons, which then activates caspases 8 and 9 and leads to neuronal apoptosis [51]. Thus the interaction of cPLA_2_α with p38 MAPK may amplify ischemic injury, as inhibition of p38 activity in the rat decreases phosphorylation of cPLA_2_α and attenuates stroke injury [7]. It is also possible that AA released by cPLA_2_α can directly stimulate phosphorylation of p38 MAPK and ERK1/2 since this has been demonstrated in cell lines [52]. Taken together this pathway interaction may potentiate early neurologic injury following MCAO.

## Conclusions

The present findings demonstrate that cPLA_2_α is an important modulator of the molecular events that occur shortly after cerebral I/R. These events are likely to amplify the cascade of inflammation, and cell death that define the process of stroke progression. Our data suggest that the late administration of a cPLA_2_α inhibitor may have limited efficacy in preventing neurologic injury produced by I/R.

## Competing interests

The authors declare that they have no competing interests.

## Authors' contributions

KK carried out all the immunomicroscopy, Western blotting (kinases), and ELISA and analysis of these and rCBF data and helped draft the manuscript. RCL carried out Western blotting (cPLA_2_α) and analysis and helped draft the manuscript. JZ carried out MCAO and drug treatments. JAK and KKK performed MCAO and measurement of rCBF. SD and RJK participated in the design of the study and helped draft the manuscript. AS conceived of the study, and participated in its design and conduct and helped draft the manuscript. All authors read and approved the final manuscript.
